# Serum spexin levels in women with diminished ovarian reserve: a prospective case–control study

**DOI:** 10.3389/fmed.2026.1917389

**Published:** 2026-08-18

**Authors:** Rumeysa Nur Ozkan, Ismail Dag, Aysegul Bestel

**Affiliations:** 1 Department of Obstetrics and Gynecology, Niksar State Hospital, Tokat, Türkiye; 2 Department of Biochemistry, Eyupsultan State Hospital, Istanbul, Türkiye; 3 Department of Obstetrics and Gynecology, Istanbul Kanuni Sultan Suleyman Training and Research Hospital, Health Sciences University, Istanbul, Türkiye

**Keywords:** anti-Müllerian hormone, biomarker, diminished ovarian reserve, infertility, neuropeptide, ovarian aging, spexin

## Abstract

**Background:**

Spexin is a recently identified neuropeptide that modulates the hypothalamic–pituitary–gonadal axis and has been shown to regulate granulosa cell function. Although circulating spexin levels decline with age and metabolic dysregulation, its relationship with ovarian reserve has not been investigated. This study aimed to evaluate serum spexin concentrations in infertile women with diminished ovarian reserve (DOR) compared with those with normal ovarian reserve (NOR).

**Methods:**

This prospective case–control study enrolled 90 infertile women (45 DOR, 45 NOR) presenting to the Infertility Outpatient Clinic at a tertiary care centre between May and October 2024. DOR was defined as serum anti-Müllerian hormone (AMH) < 1.1 ng/mL. Serum spexin was measured by ELISA on days 2–4 of the menstrual cycle. Group comparisons were performed using the independent samples *t*-test or Mann–Whitney *U* test, as appropriate. Diagnostic performance was assessed by ROC curve analysis. Correlations were evaluated using Spearman’s rank correlation coefficient.

**Results:**

Serum spexin levels were significantly lower in the DOR group than in the NOR group (*p* = 0.001). However, the DOR group was significantly older (*p* < 0.001), and after adjustment for age, the between-group difference was no longer significant (ANCOVA, *p* = 0.292). Age-stratified analysis showed no significant difference in spexin within any age band. Spexin did not independently predict DOR in multivariable logistic regression (OR = 1.00, *p* = 0.406). Positive correlations between spexin and AMH (rho = 0.375, *p* < 0.001) and antral follicle count (AFC) (rho = 0.351, *p* = 0.001) in the overall population were attenuated and lost significance after age adjustment.

**Conclusion:**

Serum spexin has not previously been evaluated in relation to ovarian reserve in women. Although spexin levels were lower in the DOR group, this difference was largely explained by age, and spexin did not independently predict DOR after age adjustment. These findings suggest that circulating spexin reflects age-related reproductive decline rather than serving as an independent marker of ovarian reserve. Age-matched studies are needed to clarify whether spexin has any DOR-specific role.

## 1 Introduction

Spexin, also known as neuropeptide Q (NPQ), is an evolutionarily conserved 14-amino acid neuropeptide first identified through bioinformatics approaches (1). Spexin belongs to the spexin/galanin/kisspeptin gene family and exerts its biological effects primarily through galanin receptor types 2 and 3 (GALR2/3) (2). Since its discovery, spexin has been implicated in a wide range of physiological processes, including energy metabolism, appetite regulation, glucose homeostasis, and cardiovascular function (3). Emerging evidence has also highlighted a role for spexin in reproductive physiology, particularly through its modulatory effects on the hypothalamic–pituitary–gonadal (HPG) axis. Spexin is expressed in gonadotropin-releasing hormone (GnRH)-expressing neurons and has been shown to suppress gonadotropin secretion in teleost and mammalian models, suggesting an inhibitory role in reproductive neuroendocrine signalling (4, 5). At the gonadal level, spexin and its receptors GALR2/3 are expressed in human granulosa cells, where spexin negatively regulates steroidogenesis and cell proliferation via MAPK, AKT, and STAT3 signalling pathways, with altered expression observed in women with polycystic ovary syndrome (PCOS) (6). Beyond humans, spexin and its receptors have been described across vertebrate reproductive tissues. In teleost fish, spexin is expressed in both the brain and the ovary and has been implicated in reproduction (7). In the human ovary, spexin and galanin receptors 2 and 3 (GALR2/3) have been localised to granulosa, theca, and cumulus cells as well as the oocyte, and *in vitro* studies have shown that spexin influences granulosa cell proliferation and steroidogenesis through GALR2/3-mediated signalling (6). Spexin and its receptors have also been examined in the hypothalamus and ovary in a letrozole-induced polycystic ovary rat model, supporting a conserved involvement of spexin in ovarian function across species (8). These findings collectively position spexin as a neuroendocrine peptide with potential relevance to ovarian function; however, its relationship with ovarian reserve has not been investigated.

Diminished ovarian reserve (DOR) refers to a reduction in the quantity and quality of oocytes in women of reproductive age, representing one of the major contributors to female infertility (9–11). The prevalence of DOR has increased in recent decades, partly due to the global trend of delayed childbearing (12). Currently, serum anti-Müllerian hormone (AMH) levels and antral follicle count (AFC) measured by transvaginal ultrasonography are considered the most reliable markers of ovarian reserve, with AMH regarded as the most sensitive single biomarker (13). However, these markers primarily reflect the size of the remaining follicle pool rather than oocyte quality, and they do not reliably predict reproductive outcomes such as pregnancy or live birth, which are largely determined by age (13). Furthermore, the molecular mechanisms underlying ovarian aging and follicular depletion remain incompletely understood, and the search for complementary serum biomarkers that may provide additional pathophysiological insight continues. Recent studies have explored novel peptide hormones and inflammatory mediators as potential indicators of ovarian function, while evidence from molecular studies suggests that processes such as oxidative stress and DNA repair deficiency may contribute to ovarian aging and reduced ovarian reserve (14–16). In this context, neuropeptides with roles in both metabolic regulation and reproductive neuroendocrine signalling represent promising yet underexplored candidates for the evaluation of ovarian reserve status.

Although spexin has been shown to modulate gonadotropin secretion and granulosa cell function, and its circulating levels are known to decline with age and metabolic dysregulation, the potential association between circulating spexin levels and ovarian reserve status remains unexplored. While recent investigations have increasingly explored genetic and molecular biomarkers in reproductive disorders within the Turkish population (17), yet novel circulating biomarkers specifically reflecting ovarian reserve remain scarce. Given the biological plausibility of spexin involvement in ovarian physiology—through its expression in GnRH neurons, its inhibitory effects on gonadotropin release, and its direct actions on granulosa cells—we hypothesised that serum spexin levels may differ between infertile women with DOR and those with normal ovarian reserve. The present study aimed to compare serum spexin concentrations between infertile women with DOR and infertile controls with normal ovarian reserve, and to explore the relationship between spexin levels and established clinical and hormonal markers of ovarian reserve.

## 2 Methods

This prospective case–control study was conducted at the Department of Obstetrics and Gynecology, Istanbul Kanuni Sultan Süleyman Training and Research Hospital, Health Sciences University, Istanbul, Turkey, between May 2024 and October 2024. The study protocol was approved by the Institutional Ethics Committee (approval number: 2024.03.59, date: 27/03/2024) and conducted in accordance with the Declaration of Helsinki. Written informed consent was obtained from all participants prior to enrollment.

Infertile women aged 20–40 years who presented to the Infertility Outpatient Clinic were eligible for enrollment. Infertility was defined as the failure to achieve a clinical pregnancy after 12 months of regular unprotected sexual intercourse in women under 35 years of age, or after 6 months in women aged 35 years or older. Participants were divided into two groups based on ovarian reserve status. As there is no universally accepted definition of DOR, the diagnosis was based on serum AMH, which is regarded as the most sensitive single marker of ovarian reserve (13). A serum AMH threshold of <1.1 ng/mL was used to define DOR, consistent with values commonly applied in the literature. AFC was defined as the total number of antral follicles measuring 2–10 mm in mean diameter in both ovaries, assessed by transvaginal ultrasonography during the early follicular phase (13). The normal ovarian reserve (NOR) group comprised women with unexplained infertility and a serum AMH level ≥1.1 ng/mL, serving as the control group. Unexplained infertility was defined as the absence of an identifiable cause following a standard evaluation, including confirmation of ovulation, tubal patency assessment by hysterosalpingography, and normal semen analysis.

Women with endometrioma, prior pelvic surgery, uterine myomas detected on transvaginal ultrasonography, irregular menstrual cycles or oligo/amenorrhea, chronic pelvic pain, type 2 diabetes mellitus requiring insulin therapy, or hypertension were excluded. All participants underwent a detailed medical history, physical examination including body mass index (BMI) calculation, speculum and bimanual pelvic examination, and transvaginal ultrasonography to evaluate the uterus, adnexa, and bilateral AFC. Serum follicle-stimulating hormone (FSH), luteinizing hormone (LH), estradiol (E2), thyroid-stimulating hormone (TSH), prolactin, and anti-Müllerian hormone (AMH) levels were measured on days 2–4 of the menstrual cycle, and midluteal progesterone was obtained to confirm ovulation. Tubal patency was assessed by hysterosalpingography, and male factor infertility was excluded through semen analysis and urological consultation.

Venous blood samples for both routine hormonal assessment and spexin measurement were collected from the antecubital vein into gel-containing biochemistry tubes on days 2–4 of the menstrual cycle. Samples were centrifuged at 4,000 rpm for 10 min, and the separated serum was stored at −80 °C until analysis. All samples were analysed in a single batch at the end of the recruitment period and underwent a single freeze–thaw cycle prior to analysis. Serum spexin concentrations were measured in duplicate using a commercially available enzyme-linked immunosorbent assay (ELISA) kit (Catalog No: 201-12-7257; Shanghai Sunred Biological Technology Co., Ltd., China) according to the manufacturer’s protocol. The intra-assay and inter-assay coefficients of variation were <10 and <12%, respectively. Optical density was measured at 450 nm using a microplate spectrophotometer (Smart Microplate Reader; USCN KIT INC.), and spexin concentrations were determined by comparison with a standard curve.

### 2.1 Statistical analysis

Statistical analyses were performed using IBM SPSS Statistics version 31.0 (IBM Corp., Armonk, NY, USA). Normally distributed continuous variables were expressed as mean ± standard deviation, while non-normally distributed variables were presented as median (interquartile range). The normality of data distribution was assessed using the Shapiro–Wilk test. Normally distributed variables were compared between groups using the independent samples *t*-test, while non-normally distributed variables were compared using the Mann–Whitney *U* test. Categorical variables were analyzed using the chi-square test. Univariate logistic regression analysis was performed for each variable separately to identify factors associated with DOR, followed by multivariable logistic regression including age, FSH, E2, and spexin to determine which factors were independently associated with DOR. To further evaluate whether the difference in spexin between groups was independent of age, an analysis of covariance (ANCOVA) was performed on log-transformed spexin values with age as a covariate. The homogeneity-of-regression-slopes assumption was assessed by testing the group × age interaction term. In addition, an age-stratified analysis was performed by comparing spexin levels between groups within predefined age bands. Receiver operating characteristic (ROC) curve analysis was conducted to evaluate the discriminative ability of serum markers for DOR, and the area under the curve (AUC) with 95% confidence intervals was calculated. The optimal cutoff value was determined using the Youden index (sensitivity + specificity − 1), with corresponding sensitivity, specificity, positive predictive value (PPV), negative predictive value (NPV), and positive likelihood ratio (LR+). Correlations between spexin levels and clinical and hormonal parameters were assessed using Spearman’s rank correlation coefficient, given the non-normal distribution of spexin values, both in the overall study population and within each group. As no prior data on serum spexin levels in women with DOR were available at the time of study design, a reliable effect size estimate could not be obtained, and a formal *a priori* power calculation was therefore not feasible. A pragmatic sample of 45 participants per group was enrolled for this initial exploratory study. A *post-hoc* power calculation was intentionally not performed, as post-hoc power is a direct function of the observed *p*-value and provides no additional information. A *p*-value <0.05 was considered statistically significant.

## 3 Results

A total of 90 infertile women were enrolled, comprising 45 in the DOR group and 45 in the NOR group. The demographic and clinical characteristics of the study population are summarised in Table 1. The DOR group was significantly older than the NOR group (*p* < 0.001), while BMI was comparable between groups (*p* = 0.847).

**Table 1:** Demographic and clinical characteristics of the study population.

Variable	NOR (*n* = 45)	DOR (*n* = 45)	*p*-value
Age (years)^b^	28.00 (25.00–30.25)	34.00 (29.00–37.00)	<0.001^*^
BMI (kg/m^2^)^b^	25.00 (22.00–27.02)	25.00 (21.90–29.10)	0.847
FSH (mIU/mL)^b^	6.55 (5.07–7.21)	8.00 (5.86–12.70)	0.001^*^
LH (mIU/mL)^b^	7.34 (5.70–11.22)	6.20 (4.70–9.26)	0.290
E2 (pg/mL)^b^	44.00 (34.75–54.50)	65.00 (44.00–148.00)	0.002^*^
AMH (ng/mL)^b^	3.00 (1.88–4.24)	0.43 (0.17–0.61)	<0.001^*^
AFC^b^	13.00 (10.75–17.25)	5.00 (4.00–6.00)	<0.001^*^
TSH (mIU/L)^a^	2.14 ± 0.92	2.13 ± 0.79	0.946
Prolactin (ng/mL)^a^	17.93 ± 7.51	15.32 ± 5.77	0.069
Spexin (pg/mL)^b^	1,310 (959–2,330)	986 (784–1,289)	0.001^*^

Data are presented as mean ± SD for normally distributed variables and as median (interquartile range) for non-normally distributed variables.

a Independent samples *t*-test.

b Mann–Whitney *U* test.

^*^p < 0.05.

Serum spexin concentrations were significantly lower in the DOR group compared with the NOR group (*p* = 0.001) (Table 1).

**Figure 1:**
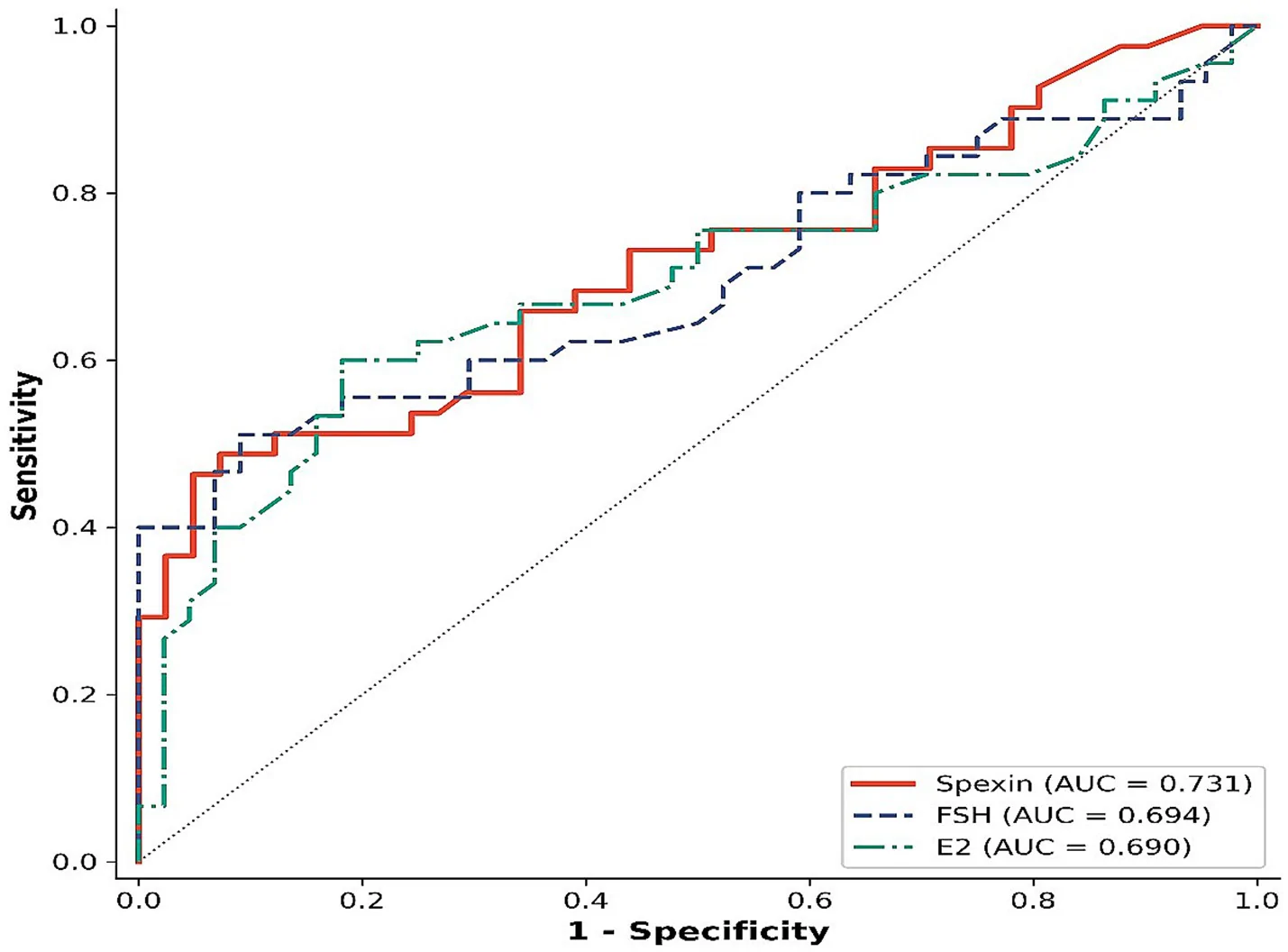
Receiver operating characteristic (ROC) curves for spexin, FSH, and E2 in discriminating DOR from NOR. AUC, area under the curve.

ROC curve analysis was performed to evaluate the discriminative ability of spexin for DOR. The AUC for spexin was 0.731 (*p* < 0.05), which exceeded those of FSH (AUC = 0.694) and E2 (AUC = 0.690). At a cutoff value of ≤920 pg/mL, sensitivity was 56.7%, specificity was 93.3%, and the positive likelihood ratio was 7.00 (Table 2, Figure 1).

**Table 2:** ROC curve analysis for the discriminative ability of serum markers for DOR.

Marker	Cutoff	AUC (95% CI)	Sensitivity (%)	Specificity (%)	PPV (%)	NPV (%)	LR(+)
Spexin	≤920 pg/mL	0.731 (0.628–0.819)	56.67	93.33	87.50	63.60	7.00
FSH	≥8.00 mIU/mL	0.694 (0.588–0.787)	51.11	90.91	85.19	64.52	5.62
E2	≥58.00 pg/mL	0.690 (0.584–0.783)	60.00	81.82	77.14	66.67	3.30

Optimal cutoff values were determined using the Youden index. AUC, area under the curve; CI, confidence interval; PPV, positive predictive value; NPV, negative predictive value; LR(+), positive likelihood ratio.

In univariate logistic regression age (OR = 1.31, 95% CI: 1.16–1.48, *p* < 0.001), FSH (OR = 1.44, 95% CI: 1.15–1.80, *p* = 0.002), and E2 (OR = 1.01, 95% CI: 1.00–1.02, *p* = 0.013) were significantly associated with DOR, whereas spexin was not (OR = 1.00, 95% CI: 1.00–1.00, *p* = 0.077). In the multivariable model, age (OR = 1.25, 95% CI: 1.06–1.46, *p* = 0.006), FSH (OR = 1.96, 95% CI: 1.30–2.93, *p* = 0.001), and E2 (OR = 1.02, 95% CI: 1.00–1.03, *p* = 0.048) remained independently associated with DOR, whereas spexin did not (OR = 1.00, 95% CI: 1.00–1.00, *p* = 0.406) (Table 3).

**Table 3:** Univariate and multivariable logistic regression analysis for factors associated with diminished ovarian reserve.

Variable	Univariate		Multivariable	
	OR (95% CI)	*p*	OR (95% CI)	*p*
Age (years)	1.31 (1.16–1.48)	<0.001^*^	1.25 (1.06–1.46)	0.006^*^
FSH (IU/L)	1.44 (1.15–1.80)	0.002^*^	1.96 (1.30–2.93)	0.001^*^
E2 (pg/mL)	1.01 (1.00–1.02)	0.013^*^	1.02 (1.00–1.03)	0.048^*^
Spexin (pg/mL)	1.00 (1.00–1.00)	0.077	1.00 (1.00–1.00)	0.406

^*^p < 0.05.

OR, odds ratio; CI, confidence interval; FSH, follicle-stimulating hormone; E2, estradiol.

To assess whether the observed difference in spexin was independent of age, an ANCOVA was performed on log-transformed spexin values with age as a covariate. After adjustment for age, the between-group difference in spexin was no longer statistically significant (*p* = 0.292). The group × age interaction was not significant (*p* = 0.234), confirming that the homogeneity-of-slopes assumption was satisfied.

In the age-stratified analysis, no statistically significant difference in spexin levels between the DOR and NOR groups was observed within any age band (20–29 years, *p* = 0.871; 30–34 years, *p* = 0.130). The 35–40 year band was not compared owing to the small number of NOR participants in this age range (*n* = 2) (Table 4).

**Table 4:** Age-stratified comparison of serum spexin levels between the DOR and NOR groups.

Age group (years)	NOR, median (IQR), *n*	DOR, median (IQR), *n*	*p*-value
20–29	1,407 (960–3,188), *n* = 28	1,525 (1032–2,185), *n* = 12	0.871
30–34	1,158 (1013–1,575), *n* = 11	889 (859–1,135), *n* = 11	0.130
35–40	1,654 (1301–2007), *n* = 2	806 (737–976), *n* = 18	—

Spexin values are expressed in pg/mL as median (interquartile range). Group comparisons were performed using the Mann–Whitney *U* test. The 35–40 year band was not compared owing to the small number of NOR participants (*n* = 2). IQR, interquartile range; DOR, diminished ovarian reserve; NOR, normal ovarian reserve.

Correlation analysis was performed using Spearman’s rank correlation given the non-normal distribution of spexin values. Spexin showed a significant positive correlation with AMH (rho = 0.375, *p* < 0.001) and AFC (rho = 0.351, *p* = 0.001) in the overall study population. The negative correlation between spexin and age was also significant (rho = −0.405, *p* < 0.001). A trend toward a negative correlation was observed between spexin and TSH (rho = −0.200, *p* = 0.071). No significant correlations were found between spexin and BMI, FSH, LH, E2, or prolactin (Table 5).

**Table 5:** Spearman correlation analysis between spexin and clinical parameters.

Variable	All patients		NOR		DOR	
	Rho	*p*	Rho	*p*	Rho	*p*
Age	−0.405	<0.001^*^	−0.000	0.999	−0.422	0.006^*^
BMI	−0.048	0.671	0.068	0.674	−0.168	0.295
FSH	−0.102	0.360	−0.005	0.973	0.026	0.874
LH	−0.005	0.968	0.096	0.551	−0.183	0.251
E2	−0.193	0.083	0.070	0.666	−0.203	0.203
AMH	0.375	<0.001^*^	−0.030	0.854	0.230	0.149
AFC	0.351	0.001^*^	−0.053	0.741	0.140	0.381
TSH	−0.200	0.071	−0.281	0.076	−0.246	0.120
Prolactin	−0.022	0.846	−0.261	0.099	0.026	0.870

^*^p < 0.05.

NOR, normal ovarian reserve; DOR, diminished ovarian reserve; BMI, body mass index; FSH, follicle-stimulating hormone; LH, luteinizing hormone; E2, estradiol; AMH, anti-Müllerian hormone; AFC, antral follicle count; TSH, thyroid-stimulating hormone.

## 4 Discussion

To our knowledge, the relationship between circulating spexin and ovarian reserve has not previously been investigated. Serum spexin levels were lower in infertile women with DOR than in those with normal ovarian reserve; however, the DOR group was significantly older, and the difference did not persist after adjustment for age. Similarly, although spexin correlated with AMH and AFC in the overall population, these correlations were attenuated and lost significance after age adjustment.

The biological plausibility of spexin involvement in ovarian reserve regulation is supported by accumulating evidence from both neuroendocrine and gonadal studies. Neuropeptides, including spexin, have been increasingly recognized for their roles in both affective and reproductive regulation (18). Spexin is expressed in GnRH-expressing neurons and magnocellular hypothalamic nuclei and acts as an inhibitory modulator of gonadotropin secretion via GALR2/3 signalling (5, 19, 20). A comprehensive review by Chen et al. highlighted that spexin suppresses LH and FSH release across multiple vertebrate species, including cichlid fish where spexin inhibits both FSH and LH through a specific galanin receptor positioning it as a key regulator within the HPG axis. At the ovarian level, Kurowska et al. (6) demonstrated that spexin and its receptors are expressed in human granulosa cells and that spexin negatively regulates both steroidogenesis and cell proliferation through MAPK, AKT, and STAT3 pathways (4). In the present study, positive correlations between spexin and both AMH and AFC were observed in the overall study population; however, these correlations did not remain significant within either group when analysed separately, and were substantially attenuated after adjustment for age. This pattern indicates that the pooled correlations were largely driven by the age-related differences between groups rather than by a direct within-group biological relationship, and should therefore be interpreted with caution. Accordingly, while these findings are consistent with the established role of spexin in granulosa cell physiology, the present data do not provide direct evidence that circulating spexin reflects the functional status of the granulosa cell pool.

The finding of reduced spexin levels in a reproductive disorder is consistent with previous reports in other clinical contexts. Ilhan et al. reported significantly lower serum spexin concentrations in women with PCOS compared with healthy controls, identifying spexin as a potential metabolic biomarker in this population (21). In male infertility, Raza et al. demonstrated that serum spexin levels were lower in men with altered sperm parameters compared with fertile controls (22). Beyond reproductive disorders, circulating spexin has been shown to decline with increasing age and BMI in healthy women, and to correlate negatively with obesity-related metabolic parameters (23–26). Notably, in the present study, no significant correlation was found between spexin and BMI in either group, and BMI did not differ between the DOR and NOR groups, indicating that metabolic factors were unlikely to account for the group difference in spexin; rather, as shown above, age was the principal explanatory factor.

In the ROC analysis, spexin showed a numerically higher AUC than FSH and E2; however, this comparison has limited clinical relevance. AMH and AFC, the current reference standards for ovarian reserve assessment, were used to define DOR status in this study and therefore could not serve as fair comparators, as their near-perfect discriminative performance is an inevitable consequence of the study design rather than a reflection of true diagnostic value. More importantly, the sensitivity of spexin at the optimal cutoff was only 56.7%, meaning that nearly half of DOR cases would be missed. Such low sensitivity, despite high specificity, precludes the use of spexin as a standalone screening or confirmatory test for DOR. Given that DOR is already defined by AMH and AFC, and that age adjustment abolished the independent association of spexin with DOR, spexin does not appear to add diagnostic value to current ovarian reserve markers. The search for novel biomarkers to complement AMH and AFC remains an active area of research, but candidate markers must demonstrate value independent of age and existing reference standards (27, 28).

The significant age difference between the DOR and NOR groups represents an important consideration in interpreting the present findings. Spexin levels were negatively correlated with age in the overall study population, and in the multivariable logistic regression model, spexin did not retain independent predictive value for DOR. This observation raises two possible interpretations that are not mutually exclusive. First, spexin may decline as a direct consequence of ovarian aging, paralleling the reduction in granulosa cell mass and follicular reserve that occurs with advancing age (29, 30). In this scenario, reduced spexin would reflect the same biological process captured by AMH and AFC, functioning as a surrogate marker of reproductive aging. Second, spexin may decline independently of ovarian reserve through age-related neuroendocrine changes, given its widespread expression in the hypothalamus and central nervous system. Distinguishing between these possibilities was not achievable in the present study due to the inherent clinical reality that DOR is more prevalent among older women, making age-matched recruitment challenging (31). When the difference in spexin was formally tested with adjustment for age using ANCOVA, the between-group difference was no longer significant, and age-stratified analysis showed no significant spexin difference within any age band. In the multivariable model, age and FSH remained independently associated with DOR, whereas spexin did not, further indicating that the crude difference in spexin was driven by the age imbalance between groups rather than by ovarian reserve itself. Together with the loss of independent predictive value in multivariable logistic regression, these findings indicate that the lower spexin levels observed in the DOR group are largely explained by the older age of this group rather than by diminished ovarian reserve per se. Spexin therefore appears to reflect age-related neuroendocrine and reproductive decline rather than functioning as an independent marker of ovarian reserve status ovarian ageing is itself a multifactorial process involving mechanisms such as oxidative stress and impaired DNA repair, which are also implicated in infertility-associated conditions such as endometriosis; whether spexin relates to these pathways remains to be explored (14, 15, 32).

A non-significant trend toward a negative correlation between spexin and TSH was observed. As this did not reach statistical significance and all participants were euthyroid, it is not interpreted further; the relationship between spexin and thyroid function would require dedicated investigation (33, 34).

This study has several limitations that should be acknowledged. First, the sample size was relatively small with 45 participants per group from a single centre, which limits the generalisability of the findings. Second, the significant age difference between the DOR and NOR groups, although reflecting the clinical reality of DOR epidemiology, precluded definitive conclusions regarding the age-independent contribution of spexin. In addition, the control group comprised women with unexplained infertility rather than healthy fertile women, which may have influenced the observed spexin levels and limits generalisability to normal fertility. Third, absolute spexin concentrations may vary between different ELISA assays; therefore, within-study comparisons rather than absolute values were emphasized. As serum spexin has not previously been measured in infertile women stratified by ovarian reserve, no directly comparable reference data exist; previously reported values were obtained in different populations using different ELISA platforms, and direct comparison of absolute values is therefore not appropriate. Fourth, the cyclic variation of spexin across the menstrual cycle in humans remains unknown; although all samples were collected during the early follicular phase to standardise the hormonal milieu, the optimal timing for spexin measurement has yet to be established. Fifth, PCOS was not formally excluded using the Rotterdam criteria; however, women with irregular menstrual cycles or oligo/amenorrhea were not enrolled, which likely excluded the majority of PCOS cases, and the low AMH levels defining the DOR group are not characteristic of polycystic ovarian morphology. Sixth, participants had not undergone IVF cycles, preventing the evaluation of spexin as a predictor of clinical outcomes such as oocyte yield or pregnancy rates. Finally, this study measured spexin only in peripheral blood; follicular fluid concentrations may more accurately reflect the local ovarian environment. Several factors known to influence circulating spexin—particularly physical activity, glucose metabolism and insulin resistance, and nutritional or fasting status at the time of sampling—were not systematically assessed, and their potential confounding influence cannot be excluded.

Despite these limitations, the present study has notable strengths, including its prospective design, standardised blood sampling during the early follicular phase, and the inclusion of well-defined DOR and NOR groups with comprehensive hormonal profiling. To the best of our knowledge, spexin has not previously been evaluated in the context of ovarian reserve, thereby opening a novel avenue for research at the intersection of neuropeptide biology and reproductive aging. Future studies should aim to validate these findings in multicentre, age-matched cohorts with larger sample sizes. Longitudinal studies evaluating spexin levels across the menstrual cycle and at multiple time points during ovarian stimulation could elucidate the dynamic regulation of spexin in relation to follicular development. Additionally, incorporating spexin into IVF protocols to assess its predictive value for oocyte quality, embryo development, and clinical pregnancy outcomes would be of significant translational relevance. Investigation of the molecular mechanisms linking spexin to granulosa cell function, including its interaction with the GALR2/3 signalling cascade in the human ovary, remains an important area for future exploration.

## 5 Conclusion

Although serum spexin levels were lower in women with DOR, this difference was largely attributable to age, and spexin did not independently predict DOR after age adjustment. Circulating spexin therefore appears to reflect age-related reproductive decline rather than functioning as an independent marker of ovarian reserve, and does not add diagnostic value to established markers. Its relationship with reproductive ageing may nonetheless warrant further mechanistic investigation. Age-matched studies, ideally incorporating follicular fluid measurements, are needed to clarify whether spexin has any ovarian reserve-specific role.

## Data Availability

The raw data supporting the conclusions of this article will be made available by the authors, without undue reservation.
